# DNA Area and NETosis Analysis (DANA): a High-Throughput Method to Quantify Neutrophil Extracellular Traps in Fluorescent Microscope Images

**DOI:** 10.1186/s12575-018-0072-y

**Published:** 2018-04-01

**Authors:** Ryan Rebernick, Lauren Fahmy, Christopher Glover, Mandar Bawadekar, Daeun Shim, Caitlyn L. Holmes, Nicole Rademacher, Hemanth Potluri, Christie M. Bartels, Miriam A. Shelef

**Affiliations:** 1 Department of Medicine, University of Wisconsin, Madison, WI USA; 2 Department of Pathology & Laboratory Medicine, University of Wisconsin, Madison, WI USA; 3 William S. Middleton Memorial Veterans Hospital, Madison, WI USA

**Keywords:** Neutrophil extracellular trap (NET), Quantification, DNA area, Fluorescence microscopy, ImageJ, Semi-automated

## Abstract

**Background:**

Neutrophil extracellular traps (NETs), extracellular structures composed of decondensed chromatin and antimicrobial molecules, are released in a process called NETosis. NETs, which are part of normal host defense, have also been implicated in multiple human diseases. Unfortunately, methods for quantifying NETs have limitations which constrain the study of NETs in disease. Establishing optimal methods for NET quantification holds the potential to further elucidate the role of NETs in normal and pathologic processes.

**Results:**

To better quantify NETs and NET-like structures, we created DNA Area and NETosis Analysis (DANA), a novel ImageJ/Java based program which provides a simple, semi-automated approach to quantify NET-like structures and DNA area. DANA can analyze many fluorescent microscope images at once and provides data on a per cell, per image, and per sample basis. Using fluorescent microscope images of Sytox-stained human neutrophils, DANA quantified a similar frequency of NET-like structures to the frequency determined by two different individuals counting by eye, and in a fraction of the time. As expected, DANA also detected increased DNA area and frequency of NET-like structures in neutrophils from subjects with rheumatoid arthritis as compared to control subjects. Using images of DAPI-stained murine neutrophils, DANA (installed by an individual with no programming background) gave similar frequencies of NET-like structures as the frequency of NETs determined by two individuals counting by eye. Further, DANA quantified more NETs in stimulated murine neutrophils compared to unstimulated, as expected.

**Conclusions:**

DANA provides a means to quantify DNA decondensation and the frequency of NET-like structures using a variety of different fluorescent markers in a rapid, reliable, simple, high-throughput, and cost-effective manner making it optimal to assess NETosis in a variety of conditions.

**Electronic supplementary material:**

The online version of this article (10.1186/s12575-018-0072-y) contains supplementary material, which is available to authorized users.

## Background

Neutrophil extracellular traps (NETs) consist of extracellular decondensed chromatin affixed with anti-microbial proteins [1] and are created by neutrophils in a process called NETosis. Although the primary roles of NETs and similar structures are thought to be immobilization and destruction of invading microorganisms, NETs have also been implicated in thrombosis as well as many diseases including systemic lupus erythematosus, rheumatoid arthritis, antiphospholipid antibody syndrome, vasculitis, pancreatitis, and type I and type II diabetes [2–10]. For example, NETs are increased in rheumatoid arthritis, antiphospholipid antibody syndrome, and vasculitis and they contain proteins targeted by autoimmune antibodies in rheumatoid arthritis and vasculitis [11–13]. Also, NETs are required for thrombosis in murine models of both antiphospholipid antibody syndrome and deep vein thrombosis [10, 14] and NETs drive pancreatitis by ductal occlusion [2]. Given the numerous ways by which NETs exacerbate human disease, clinical biomarkers and treatments are being developed that detect and inhibit NETs [15]. However, NETs can be challenging to study and quantify. Given limitations in the methodology for NET quantification, many unanswered questions remain about how NETs may contribute to disease. Establishing efficient and accurate methods for quantifying NETosis under a variety of experimental conditions holds the potential to further elucidate the role of NETs and similar structures in normal and pathologic processes.

Scientists have used a variety of methods to study the function of NETs (i.e. microbial killing assays [16], complement activation assays [17], detection of interactions with other cells and structures [18]) as well as to quantify NETs. Methods for quantifying NETs and NET-like structures have utilized a wide variety of techniques including enzyme-linked immunosorbent assay (ELISA), flow cytometry, cell-free DNA-based assays, 3-dimensional confocal microscopy, and fluorescence microscopy [11, 19–23]. Although each of these methods has strengths, method-specific limitations exist [24]. One of the most commonly used methods to quantify NETosis is to count by eye the number of cells that have decondensed DNA and/or NET-associated proteins as visualized by fluorescence microscopy [25–27]. While counting NETs and NET-like structures by eye has the advantage of direct visualization, quantifying these images by eye is time consuming, subjective, and can be inconsistent between samples, over time, and across individuals.

Semi-automated methods of quantification can reduce unintentional bias, inconsistency, and time requirements. While semi-automated methods exist for quantification of NETs identified with one or two fluorescent markers and either confocal or wide field microscopy [2, 19, 28–30], each method faces obstacles related to the cost of the confocal microscope, information provided only in average and not for individual cells, lack of cut-offs to identify NETs, and/or limited ability to exclude nuclear fragments and overlapping cells, i.e. multiples. Additionally, these programs limit the user to specific markers and can be challenging for new users to install and to operate.

To advance the quantification of DNA area and NETosis and to provide a simple to use and low cost approach, we created DANA (DNA Area and NETosis Analysis) to analyze fluorescently stained neutrophils in standard fluorescent microscope images. DANA is an open-source ImageJ/Java-based program that utilizes a single fluorescence channel to accurately and efficiently quantify DNA area and NET-like structures on a per cell, per image, and per sample basis while consistently excluding overlapping cells and fragments.

## Methods

### Human Subjects

Subjects 18 years or older receiving primary care and rheumatology care (for rheumatoid arthritis subjects) at UW Health were recruited and informed consent was obtained after the nature and possible consequences of the studies had been fully explained. Rheumatoid arthritis subjects were initially identified as individuals with two or more outpatient visits with any provider with rheumatoid arthritis associated ICD codes (ICD-9 codes 714.0–714.33, 714.9 or any ICD-10 code starting with M05, M06, or M08) within 24 months [31] or one visit and a positive anti-CCP test. Rheumatoid arthritis subjects were confirmed to have rheumatoid arthritis based on manual review of the three most recent rheumatologist progress notes in the electronic medical record. Control subjects were identified in primary care clinics or by snowball recruiting. Controls were excluded if they had any of the following diagnoses as determined by verbal screen and manual review of the medical record: rheumatoid arthritis or other autoimmune or inflammatory diseases including but not limited to lupus, Sjogren’s Syndrome, scleroderma, multiple sclerosis, type I diabetes, psoriasis or psoriatic arthritis, ankylosing spondylitis, reactive arthritis, ulcerative colitis, Crohn’s disease, cancer of the blood cells including leukemia or lymphoma.

### Animals

Wild-type adult mice (both male and female) that were either on a DBA/1 J or C57BL/6 background (The Jackson Laboratory, Bar Harbor, USA) were used for this study. Mice were maintained in pathogen-free conditions.

### Human Neutrophil Isolation and Stimulation

Human blood samples were collected into EDTA tubes and neutrophils were purified using the EasySep™ Direct Neutrophil Isolation Kit (STEMCELL Technologies, Vancouver, Canada) according to the manufacturer’s instructions. Neutrophils were determined to be 95% pure by flow cytometry. Following purification, 2.5–5 × 10^4^ neutrophils were pipetted onto acid-washed coverslips coated with poly-L-lysine (Sigma Aldrich, St. Louis, USA) and then incubated in Roswell Park Memorial Institute (RPMI) 1640 medium for either 2 or 4 h at 37 °C, 5% CO_2_ followed by fixation in 70% ethanol overnight at 4 °C. Then, the neutrophils were washed with phosphate buffered saline (PBS, Corning, Corning, USA), stained with 250 nM Sytox (ThermoFisher Scientific, Waltham, USA) and washed with PBS again. Coverslips were mounted onto microscope slides with Prolong Diamond Antifade (ThermoFisher Scientific), allowed to dry, sealed with clear nail polish (Sally Hansen, New York, USA), and stored at -80 °C.

### Mouse Neutrophil Isolation and Stimulation

Murine neutrophils were isolated and stimulated as previously reported [25]. Briefly, neutrophils were purified from murine bone marrow, plated on poly-L-lysine coated coverslips and incubated for 4 h at 37 °C, 5% CO_2_ either without treatment or with 10 μg/ml lipopolysaccharide (LPS) +/− 100 ng/ml TNF-α. Cells were then fixed, permeabilized, and stained with 4′,6-diamidino-2-phenylindole dihydrochloride (DAPI) as previously [25] followed by mounting and storage as above. Of note, neutrophils were determined to be 95% pure by flow cytometry.

### Microscope Images

All images were obtained using a Leica DM4000 B upright fluorescent microscope (Wetzlar, Germany) equipped with a Leica EL6000 light source and a Retiga 4000R camera (Qimaging, Surrey, Canada) running Image-Pro Plus software version 6.3.0.512 (Media Cybernetics, Rockville, USA). Human neutrophil images were captured at a magnification of 200× and murine neutrophils images were captured at 400×. Five images were taken per coverslip at standardized locations. If no cells were visible within one of the locations, additional images were taken at points exactly between the standard five locations and labeled accordingly.

### DNA Area and NETosis Analysis (DANA) Design and Standardization

**Fig. 1:**
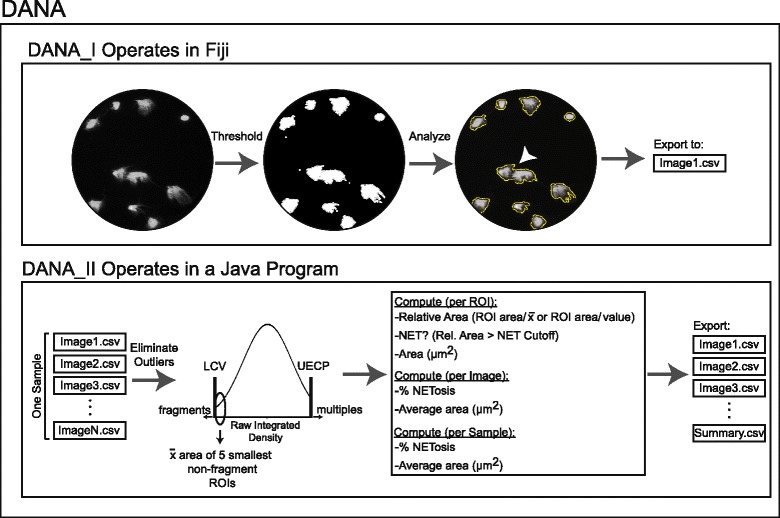
DANA operates in two parts to provide information on NETosis and cellular DNA area. In DANA_I, images are loaded into Fiji where they are thresholded, analyzed, and information on each region of interest (ROI) is outputted to a .csv file for each image of the sample. Once all images for a single sample have been processed within DANA_I, DANA_II then loads the ROIs from all .csv files from that sample. ROIs with raw integrated densities smaller than the lower cutoff value (LCV) or larger than the mean plus the product of the upper elimination cutoff parameter (UECP) and the sample standard deviation are excluded to eliminate fragments and multiples. Of the remaining ROIs, the average area of the five smallest ROIs is computed (denoted as x-bar). The five smallest non-excluded ROIs are considered the most condensed nuclei. The areas of all remaining ROIs are divided by x-bar (or by a user defined value if required by future users) and ROIs are labelled as NETs if their relative area is greater than the NET cutoff. Data on NETs and DNA area is exported to an image-specific .csv file. These image-specific files also contain information on the percent NETosis and average cellular DNA area for that specific image. Additionally, a summary.csv file is exported with information on the percent NETosis and average cellular DNA area for the entire sample. Outlines in the rightmost circle indicate ROIs. The arrowhead in the rightmost circle indicates multiple cells present within a single ROI

DANA operates in two components – the first taking place in ImageJ [32] (DANA_I) and the second in a Java program (DANA_II) (Fig. 1). The ImageJ portion serves to quantify the area, raw integrated density, aspect ratio, roundness, maximum and minimum brightness, and solidity of each region of interest (ROI). A single ROI is intended to contain a single cell, but can also contain cell fragments or multiple cells if there is overlap. DANA achieves optimal accuracy by processing all of the images for a sample together. For this study, a human sample is defined as all images obtained for a single human subject at all time points. A single mouse sample contains all images from a single mouse including unstimulated and stimulated treatment groups.

To generate the ROIs and their associated information, all of the images for a specific sample are opened into a fully-loaded version of ImageJ called Fiji [33], and DANA_I is run on each image individually. DANA_I converts the image to 8-bit grayscale, then thresholds the image and analyzes the particles generating ROIs with areas between 20 μm^2^ and 3500 μm^2^. Repeat analyses indicated that ROIs smaller than 20 μm^2^ were typically fragments and ROIs larger than 3500 μm^2^ typically contained multiple cells. The “analyze particle” function is used to export information on each ROI into a .csv file and displays the original image overlaid with the numerically labeled ROI outlines. To ensure accuracy, the user manually eliminates any images for which DANA_I generates seven or more ROIs containing multiple cells or does not accurately capture cells visualized on screen, for example if extremely high background interferes with DANA. If the sample’s images are known to contain less than 7 overlapping cells per image, DANA_I can be run in a batch processing mode, where all images from a single sample are processed automatically together.

DANA_II automatically imports all of the Fiji-generated .csv files from the sample, eliminates ROIs corresponding to cell fragments or multiples, and exports a .csv file containing information on the DNA area and NET formation for that sample. To accomplish this, DANA_II pools the ROIs from all of the .csv files from the sample and eliminates those with raw integrated densities outside of the standardized cutoffs (see below). The areas for the remaining ROIs are then calculated relative to the areas of the five smallest remaining ROIs. These five smallest ROIs represent condensed nuclei for the sample. Thus, it is imperative that for any sample, a negative control is included with condensed nuclei. Any ROIs not previously excluded that have a relative area above the NET cutoff (see below) are labeled as a NET-like structure. DANA_II then exports a .csv file for each image which contains information on the area, relative area compared to condensed nuclei, and NET status for each ROI within that image. This .csv file also contains information on the number and percentage of ROIs above the NET cutoff value, the percentage of NETosis, and the average cellular DNA area for that specific image. In addition, for each sample, the program exports a summary .csv file, containing information on the sample’s average percentage NETosis and DNA area.

To generate the cutoffs, 15 images were analyzed by eye to count the total cell number, the number of overlapping cells, and the number of NET-like structures. DANA was then run on these images using different cut-off values. The cut-off values which gave the most accurate count of NETs as well as exclusion of multiples and fragments were selected for each sample type (i.e. human Sytox-stained and murine DAPI-stained neutrophils). We determined that ROIs with raw integrated densities greater than 1.5 standard deviations from the mean were typically multiples and ROIs with raw integrated densities less than 20,000 were fragments for Sytox-stained human neutrophils. Also for Sytox-stained human neutrophils, a threshold of 4.70× the area of the smallest five cells was optimal to accurately identify NET-like structures. For DAPI-stained murine neutrophils, ROIs with raw integrated densities above 2.0 standard deviations from the mean were typically multiples and ROIs with raw integrated densities less than 10,000 were often fragments. A threshold of 5.34× the area of the smallest five cells was optimal to accurately identify NETs and NET-like structures in DAPI-stained murine neutrophils. These cut-offs were then applied to images not previously analyzed by DANA and accuracy was confirmed by eye. As final validation steps, the murine and human neutrophils were confirmed to contain myeloperoxidase (MPO, Additional file 1: Fig. S1) and NET-like structures were confirmed to be extracellular structures (Sytox staining of live cells) with decondensed chromatin (DAPI and citrullinated histone staining) and anti-microbial molecules (MPO staining) as shown in Additional file 2: Fig. S2. DANA, as well as instructions and materials to install and optimize DANA’s cut-offs for a future user’s specific requirements, can be found at the Shelef Lab website: http://www.medicine.wisc.edu/rheumatology/shelef-lab.

### Statistics

**Fig. 2:**
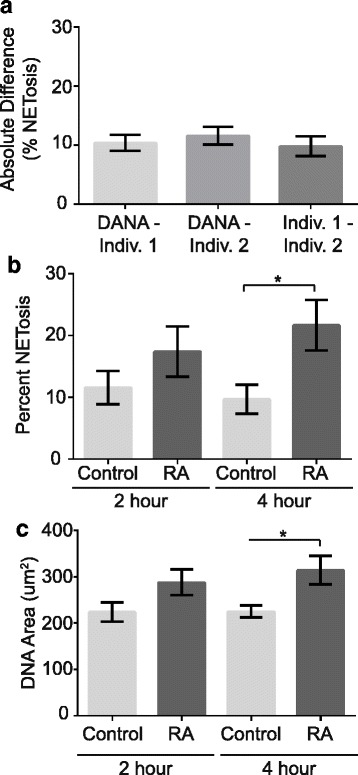
Detection of Sytox-stained NETs by DANA in rheumatoid arthritis subjects and controls. Neutrophils from rheumatoid arthritis (RA) subjects and controls were incubated for 2 and 4 h on coverslips, then fixed, stained with Sytox, mounted, and imaged at 200×. **a**. Two individuals independently counted the number of nuclei and NET-like structures by eye and then DANA was used to calculate the percent NETosis for all images (*n* = 78 images). The mean absolute differences with SEM for percent NETosis per image for DANA versus individual 1 by eye, DANA versus individual 2 by eye, and individual 1 versus individual 2 by eye were graphed. DANA was then used to quantify percent NETosis (**b**) and DNA area (**c**) with average and SEM graphed for RA versus control neutrophils (2 h RA, *n* = 24 subjects; 2 h control, *n* = 11 subjects; 4 h RA, *n* = 21 subjects; 4 h controls, *n* = 17 subjects; *p < 0.05)

**Fig. 3:**
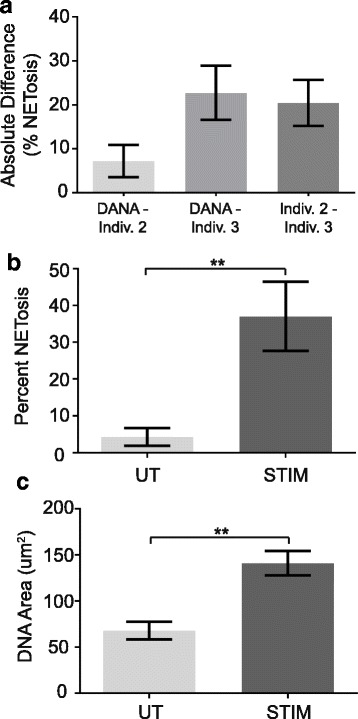
Detection of DAPI-stained murine NETs by DANA. Murine neutrophils were either left untreated (UT) or stimulated (STIM) for 4 h to induce NETosis on coverslips, then fixed, stained with DAPI, mounted, and imaged at 400×. **a**. Two individuals independently counted the number of nuclei and NET-like structures by eye and then DANA was used to calculate the percent NETosis for all images (*n* = 12 images). The mean absolute difference with SEM for percent NETosis per image was graphed for DANA versus individual 2 by eye, DANA versus individual 3 by eye, and individual 2 versus individual 3 by eye. DANA was used to quantify percent NETosis (**b**) and DNA area (**c**) with average and SEM graphed for unstimulated and stimulated murine neutrophils (*n* = 5 mice). For all panels, **p < 0.01

Differences between rheumatoid arthritis subject versus control neutrophils and stimulated versus unstimulated murine neutrophils were analyzed using an unpaired *t* test (Figs. 2B, 2C, 3B, 3C). To compare absolute differences in human and mouse neutrophil quantification, a one-way ANOVA with Tukey’s multiple comparisons test and a single pooled variance was used (Figs. 2A and 3A). Statistical analyses were performed using GraphPad Prism software version 6.04 for Windows (La Jolla, USA).

## Results

Following standardization, we determined if DANA could quantify NET-like structures similarly to individuals trained to identify NETs by eye. Two individuals independently counted by eye the total number of NET-like structures (defined as large, cloud-like DNA masses) and cells per image of in vitro cultured, Sytox-stained human neutrophils for 78 images captured at 200× amounting to over 2400 neutrophils from 9 subjects. Individual 1 calculated 26.52% and individual 2 calculated 26.18% of the cells from these 78 images had undergone NETosis. After the counts by eye were complete, DANA calculated a similar 21.35% NETosis for the images. In addition to total percent NETosis, we calculated the average absolute difference in NET-like structures per image quantified by DANA versus each individual, as well as between individuals in order to identify significant differences between DANA and counts by eye. As shown in Fig. 2A, there is no significant difference between DANA and counts by eye or between individuals. Further, whereas DANA took approximately 1.5 h to analyze these images, the manual process took 7 to 10 h.

Having established that DANA was capable of quantifying human NET-like structures similarly to trained individuals, we next compared the frequency of NET-like structures and average DNA area between neutrophils from rheumatoid arthritis subjects and controls. Given previous reports that neutrophils from rheumatoid arthritis subjects are more likely to form NETs than controls [9], we anticipated that DANA would generate similar findings. As shown in Fig. 2B, DANA quantified a significantly higher percent of NETosis after 4 h of incubation for rheumatoid arthritis subjects compared to controls. Additionally, DANA quantified a higher average DNA area at 4 h of incubation for rheumatoid arthritis subjects compared to controls (Fig. 2C). DNA area and percent NETosis for each subject can be found in Additional file 3: Table S1.

We then wanted to address two additional parameters. First, we wanted to ensure that DANA was simple to install and standardize. Second, we wanted to validate DANA’s applicability to other species, stains, and magnifications. To address these goals, an individual trained in identifying NET-like structures by eye, but with no programming experience (individual 2), independently installed and standardized DANA for DAPI-stained murine neutrophils imaged at 400×. Two individuals (individual 2, described above, and individual 3, who also was trained in identifying NET-like structures by eye, but with no programming experience) then independently counted NET-like structures by eye for 12 DAPI-stained murine neutrophil images and next used DANA to analyze the same images. Individual 2 calculated 12.11% NETosis by eye and individual 3 calculated 26.15% NETosis by eye, compared to 11.08% calculated by DANA. As shown in Fig. 3A, the absolute difference between percent NETosis calculated by eye versus DANA for each individual was low and did not reach statistical significance.

Next, DANA was used to analyze 40 images of murine neutrophils that were either stimulated to induce NETosis or unstimulated. The frequency of NET-like structures and average DNA area were compared for the unstimulated versus stimulated neutrophils. As shown in Fig. 3B and C, DANA calculated higher rates of NETosis and larger DNA areas in stimulated neutrophils compared to untreated neutrophils. DNA area and percent NETosis for each mouse can be found in Additional file 3: Table S2. Taken together, these data suggest that DANA can be easily installed and standardized to quantify NET-like structures in different species using different stains at different magnifications.

## Discussion

In this manuscript, we describe DANA, a simple ImageJ/Java-based program that can quantify murine and human NET-like structures in fluorescent microscope images at a frequency comparable to trained individuals quantifying NET-like structures by eye. DANA also detects, as expected, a higher frequency of NET-like structures formed by neutrophils from rheumatoid arthritis subjects compared to controls as well as more NET-like structures in stimulated compared to unstimulated murine neutrophils. Thus, DANA accurately identifies human and murine NET-like structures according to standards in the field. Furthermore, DANA can provide the DNA area for each neutrophil and demonstrate that neutrophils from rheumatoid arthritis subjects display more DNA decondensation than controls. Not only did DANA provide this information on a per cell, per image, and per sample basis, but it did so in a fraction of the time required for quantification by eye. Other methods of analyzing NETosis [19, 20, 28, 29] do not automatically characterize individual NETs on a per cell basis. Further, DANA operates in a reproducible manner that avoids unintentional bias between samples and eliminates variability between individuals. For example, individual 1 and individual 2 calculated extremely similar rates of NETosis for human neutrophils (Fig. 2A), but individual 2 and individual 3 had a trend towards greater variability between their counts (Fig. 3A). DANA eliminates the variability between individuals providing consistent and standardized counts. Also, DANA does not require exceptionally expensive equipment such as confocal microscopy [19]. Thus, utilizing basic neutrophil purification and staining techniques, standard fluorescence microscopy, and free computing platforms, DANA is readily implementable to quantify NET-like structures and DNA area.

DANA has several additional advantages as compared to other methods for analyzing NETosis. One advantage is that quantification is based on single-channel DNA-stained fluorescence images. Some NET quantification methods include co-localization of neutrophil-specific proteins such as MPO [34] or NET-specific proteins such as citrullinated histone [35]. However, such markers are not always used [30], are not always helpful, and can limit quantification. For example, MPO and similar neutrophil-specific markers are present in both resting neutrophils and NETs (Additional file 1: Fig. S1). Thus, the use of MPO in purified neutrophils adds extra steps for staining, complexity to analysis, lack of flexibility in markers, and no benefit to the quantification of NET-like structures. For mixed cell populations, MPO is helpful to identify NETs. In that situation, a future researcher could use the instructions that we provide to optimize DANA to detect NETs as defined by MPO staining or other markers if desired. Some other markers used for NET detection are not present in resting neutrophils, such as citrullinated histone. However, NET-like structures can have different cargo and thus co-localization markers may not be present on all NET-like structures [36, 37] limiting quantification. DANA’s single channel method of quantification provides simplicity of staining and analysis, inclusion of all NET-like structures, as well as the flexibility to quantify extracellular traps generated by macrophages [38], mast cells [39], monocytes [40] and eosinophils [41], which have different markers than NETs.

An additional advantage of DANA is that it addresses the limitation arising from the issue of segmenting multiple cells and eliminating cell fragments, which is incompletely addressed by other programs [28–30]. DANA uses a sample-specific threshold for raw integrated density to eliminate fragments or multiple cells gated together, a novel approach. Although DANA will occasionally fail to retain large NETs or extremely condensed nuclei for analysis in order to consistently eliminate multiples and fragments, we have found that when neutrophils are plated dilutely in order to avoid clusters of neutrophils and NETs, the number of excluded cells is small and the overall results agree with counts by eye (Figs. 2 and 3).

Two final major advantages of DANA are ease of use and flexibility. Unlike other methods that we found extremely challenging to implement [2, 28, 30], DANA is simple to install, simple to use with our standardized settings for investigators performing similar experiments, and simple to optimize if needed for experiments using different cell types, stains, or magnifications, a key innovation. Instructions for how to optimize cutoffs based on a user’s specific cell type, image magnification, stains, and other variables are freely available making DANA easy to use for a wide range of applications.

## Conclusions

DANA is an accurate, semi-automated method for quantifying DNA area and frequency of NETosis in fluorescent microscope images on a per cell, per image, and per sample basis while excluding fragments and multiples. DANA provides several advantages over other methods for quantifying NETs. It combines the simplicity of single channel fluorescence microscopy with a rapid, reliable, and non-subjective means of quantifying NETosis to eliminate variability in quantification. Further, DANA can be used as standardized in this report or it can be optimized easily for a variety of experimental conditions and cell types, a novel feature. Finally, we provide DANA in an easily accessible, open-source format with simple instructions for use.

## Additional files

Additional file 1: **Figure S1.** Immunofluorescent staining of DNA and myeloperoxidase (MPO) in neutrophils. (JPEG 3214 kb)

Additional file 2: **Figure S2.** Marker analysis of NET-like structures. (PDF 967 kb)

Additional file 3: **Tables S1** and **S2. Table S1:** DANA results for each human subject in Figs. 2B and C. **Table S2:** DANA results for each mouse in Figs. 3B and C. (PDF 63 kb)

## Abbreviations

DANA: DNA Area and NETosis Analysis
DAPI: 4′,6-diamidino-2-phenylindole dihydrochloride
NET: Neutrophil Extracellular Trap
