# Supplementary figures and images for: DNA Area and NETosis Analysis (DANA): a High-Throughput Method to Quantify Neutrophil Extracellular Traps in Fluorescent Microscope Images
Source: Biol Proced Online. 2018 Apr 1;20:7. doi: 10.1186/s12575-018-0072-y (PMC5878938; doi:10.1186/s12575-018-0072-y)

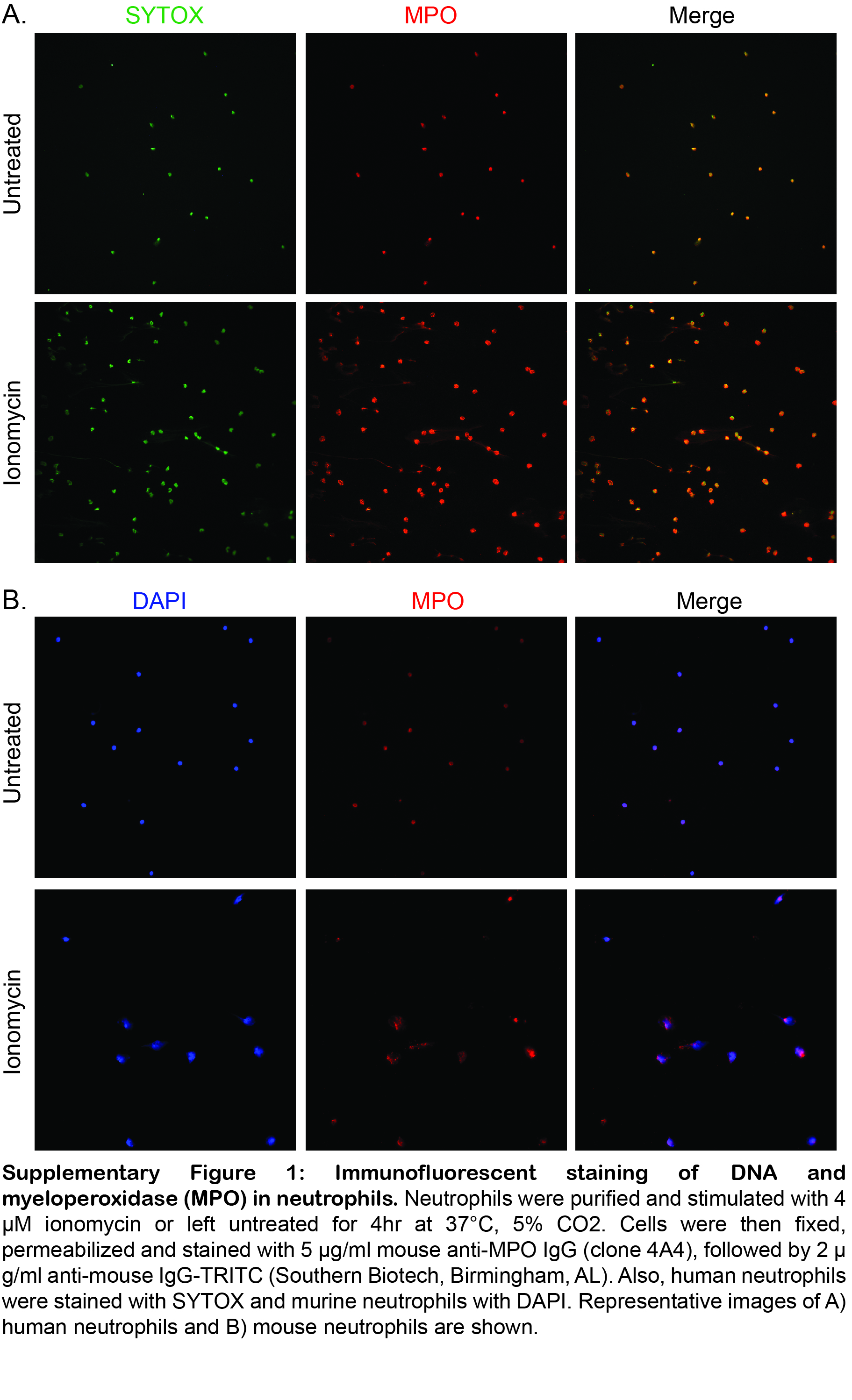

Supplement: Supplementary file 1 — Figure S1. Immunofluorescent staining of DNA and myeloperoxidase (MPO) in neutrophils. (JPEG 3214 kb) [file 12575_2018_72_MOESM1_ESM.jpg]
