# Supplementary material for: DNA Area and NETosis Analysis (DANA): a High-Throughput Method to Quantify Neutrophil Extracellular Traps in Fluorescent Microscope Images
Source: Biol Proced Online. 2018 Apr 1;20:7. doi: 10.1186/s12575-018-0072-y (PMC5878938; doi:10.1186/s12575-018-0072-y)
Supplement: Supplementary file 2 — Figure S2. Marker analysis of NET-like structures. (PDF 967 kb) [file 12575_2018_72_MOESM2_ESM.pdf]

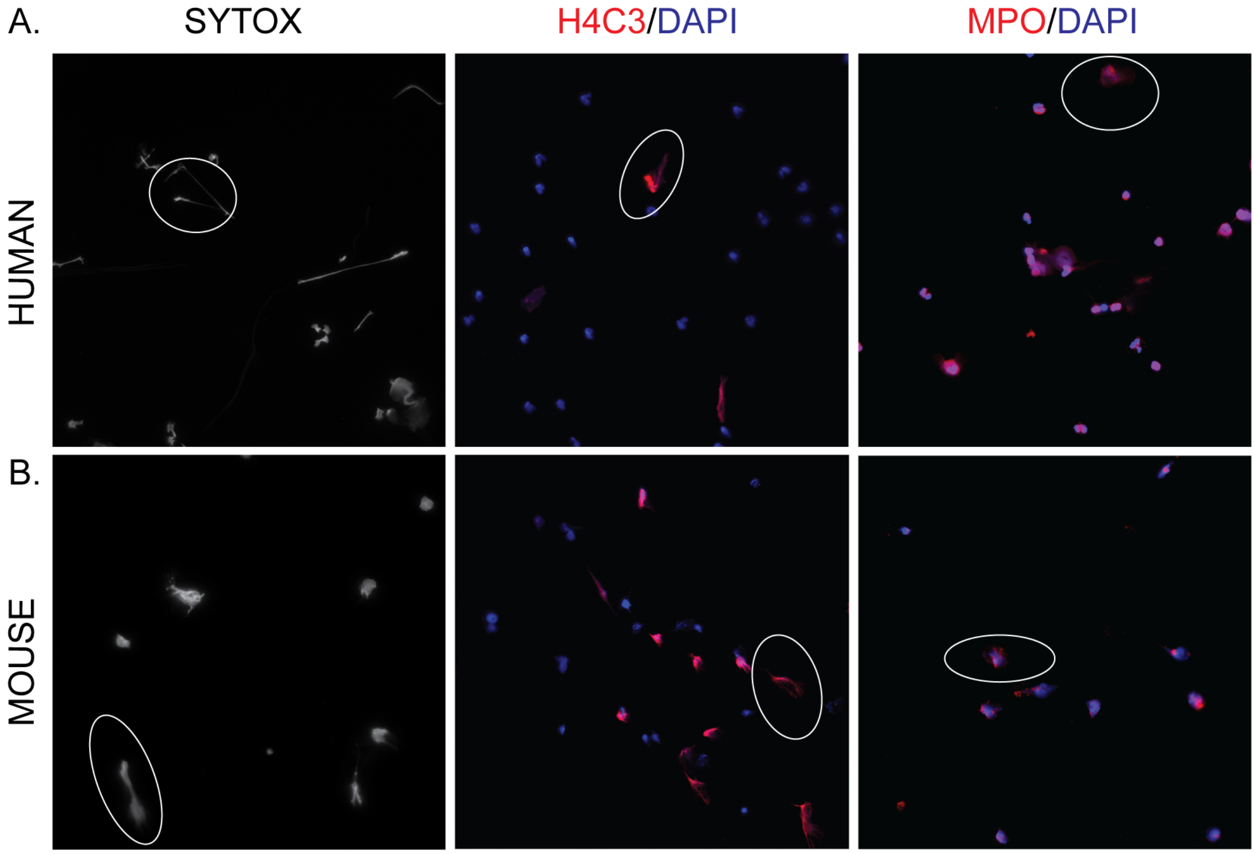

Supplementary Figure 2. Marker analysis of NET-like structures. Human and murine neutrophils were purified, plated on glass coverslips, and either incubated with ionomycin and Sytox, a cell impermeant DNA stain, to detect extracellular DNA or incubated with ionomycin alone followed by fixation, permeabilization, staining with 4',6-diamidino-2-phenylindole (DAPI) as well as either anti-citrullinated histone H4 (H4C3, a marker of decondensed chromatin in NET-like structures) or anti-myeloperoxidase (MPO, an anti-microbial molecule present in NETs). All coverslips were mounted, imaged at 400x, analyzed by DANA, and analyzed by eye for NET-like structures. Representative images of human (A) and murine (B) neutrophils stained with Sytox (left panels), DAPI in blue and anti-H4C3 in pink (center panels), and DAPI in blue and anti-MPO in pink (right panels). Circles indicate examples of cells determined to be NET-like structures by eye and by DANA. These circled structures contain extracellular DNA, decondensed chromatin with citrullinated histones, and MPO.
