# Supplementary material for: DNA Area and NETosis Analysis (DANA): a High-Throughput Method to Quantify Neutrophil Extracellular Traps in Fluorescent Microscope Images
Source: Biol Proced Online. 2018 Apr 1;20:7. doi: 10.1186/s12575-018-0072-y (PMC5878938; doi:10.1186/s12575-018-0072-y)
Supplement: Supplementary file 3 — Tables S1 and S2. Table S1: DANA results for each human subject in Figs. 2B and C. Table S2: DANA results for each mouse in Figs. 3B and C. (PDF 63 kb) [file 12575_2018_72_MOESM3_ESM.pdf]

Supplementary Table 1. DANA results for each human subject in Figure 2B and 2C.

Subject		2 hour		4 hour	
		NETosis (%)	DNA Area (μm^2)	NETosis (%)	DNA Area (μm^2)
Control	1	20.0	318.9	3.7	247.7
	2	7.3	218.7	10.2	252.2
	3	8.8	229.5	12.5	231.6
	4	8.7	196.7	0.0	151.5
	5	0.0	117.9	0.0	118.1
	6	5.2	163.0	5.6	189.6
	7	18.8	264.9	14.4	317.7
	8	28.3	320.4	8.3	191.4
	9	19.5	251.2	31.7	304.1
	10	11.1	259.9	21.9	263.9
	11	0.0	125.9		
	12			23.2	285.6
	13			0.0	192.8
	14			20.0	253.0
	15			1.9	157.1
	16			1.8	226.4
	17			0.0	236.5
	18			9.9	216.9
Rheumatoid Arthritis	1	1.3	150.2	37.9	324.6
	2	3.0	163.6	0.0	112.1
	3	57.1	587.7	13.8	363.7
	4	13.1	340.4	50.8	461.6
	5	12.1	278.0	31.4	343.8
	6	12.6	291.5	15.7	311.0
	7	3.3	174.9	70.4	612.2
	8	49.6	446.5	25.5	267.1
	9	0.0	116.9	0.0	143.0
	10	1.4	162.0	9.3	210.9
	11	2.0	203.4	2.3	195.4
	12	0.0	194.0	0.0	163.3
	13	2.0	135.9	0.0	140.8
	14	1.1	145.6	22.2	299.2
	15	20.3	257.3	35.7	364.7
	16	17.5	319.8	16.2	351.8
	17	4.6	230.7	4.6	230.7
	18	0.0	146.1		
	19	27.1	359.9		
	20	48.0	484.5		
	21	6.6	306.2		
	22	48.3	367.2		
	23	45.0	430.6		
	24	9.5	402.0		
	25	65.0	640.0		
	26	2.6	238.1		
	27			4.5	215.9
	28			26.9	388.3
	29			30.6	306.7
	30			32.7	431.3
	31			46.5	680.0

Supplementary Table 2. DANA results for each mouse in Figure 3B and 3C.

	Untreated		Stimulated	
Mouse	NETosis (%)	DNA Area (μm^2)	NETosis (%)	DNA Area (μm^2)
1	1.3	52.9	26.1	126.8
2	0.0	38.9	14.0	111.4
3	0.0	70.0	63.2	186.4
4	8.6	103.0	26.9	127.4
5	11.5	64.7	55.1	152.4
